# Fracture of the Jaws

**Published:** 1890-02

**Authors:** D. W. Fellows

**Affiliations:** Portland, Me.


### FRACTURE OF THE JAWS.[1]

BY DR. D. W. FELLOWS, PORTLAND, ME.

Mr. President and Gentlemen:—It is my purpose to-night to consider fracture of the jaws, and particularly fracture of the lower jaw; but I think it not inappropriate to present a brief review of the nature, causes, and diagnosis of fractures, the principles of treatment, and mode of cure.

Fracture is defined as a solution of continuity of the osseous tissue.

Fractures may be simple, compound, comminuted, or impacted. These terms are familiar and need no explanation.

Various diseases, by impairing the structure of the bones, act as predisposing causes of fracture. The most important of these are syphilis, cancerous diseases, osteomalacia, and rickets. But fracture of healthy bones must be always a result of violence, and nearly always of external violence, either direct or indirect, though in numerous instances bones have been broken by muscular contraction.

The reliable symptoms of simple fracture are crepitation, deformity, and preternatural mobility. The peculiar sound caused by rubbing the ends of the broken bone together, known as crepitation, is characteristic of fracture. It may generally be felt as well as heard. This symptom may be obscured by much swelling or by great depth of soft tissue, and in impacted fracture it will be absent.

Deformity is generally, but not always, present, and it may be a result of the cause which produced the injury or of the action of muscles upon the fragments.

Preternatural mobility is an important symptom of fracture, and is rarely absent unless the fracture be impacted.

The manner of repair in cases of fracture merits some consideration. The first stage, which is a period of preparation, occupies

---

[1] Read before the American Academy of Dental Science, December 4, 1889.

about one week. During this period, in favorable cases, inflammation subsides, the inflammatory products and extravasated blood are absorbed; pain, swelling, and traumatic fever disappear. From the eighth to the seventeenth or twentieth day the parts become red and injected, and covered with a layer of embryonic cells, and towards the end of this period the granulation tissue becomes more and more firm, until, finally, the tissue is converted into bone, forming, in the case of long bones, two layers, one within the medullary canal and the other encircling the ends of the fragments. This is known as the temporary or provisional callus, which is Nature's splint for holding the fragments in position until repair is rendered complete by the deposit of osseous tissue between the broken ends. The temporary callus, it may be stated, is much harder than normal bone. The final stage of repair, after the bone has been united by a sound bony tissue, consists in the absorption of the provisional callus, leaving the bone smooth and in proper condition for continued performance of its function. The whole process may occupy several months, and if the ends of the fragments are not in proper relation to each other the process may be rendered very long and tedious. In union of a fractured lower jaw the temporary callus is found to be much less than in ordinary long bones, the union being direct after the formation of a small encircling band of temporary callus.

Surgeons declare that the treatment of fractures requires a greater amount of ready knowledge, skill, and judgment than any other department of surgery, and that they always approach them with some misgiving.

Surgeons assure me, further, that fractures of the lower jaw are perhaps the most difficult of all fractures to treat successfully, or in such a manner that there shall be no subsequent impairment of function, no discomfort, and no deformity.

The diagnosis of fracture of the lower jaw is usually not difficult. Nearly always crepitation may be found, together with preternatural mobility, and, I believe, in most cases there will be some deformity, though in a single fracture of the body of the bone it may not be marked; and yet the injury, like many others, may be easily overlooked unless the attention is specially directed to it. In one case that I treated the patient was examined by two physicians at different times and no fracture discovered, although the bone was broken through the body at the right of the symphysis, and through the left ramus. The man was afterwards taken to the Maine General Hospital, and when I saw him crepitation was distinct, and the deformity and mobility of the parts quite pronounced.

The methods that have been employed for reducing and treating fractures of the jaw are almost innumerable. The various forms of bandaging, and external splints, tying the teeth with thread or wire, piercing the fragments and wiring them together, have all proved very troublesome or ineffectual, except in simple cases without displacement of the fragments.

The idea of an interdental splint is far from new, some such appliance having been used by Paré three hundred and fifty years ago. Dr. Hamilton used gutta-percha moulded to the teeth. Plates or splints of metal have also been used, but the peculiar adaptability of vulcanized rubber for the purpose seems to have been first recognized by Dr. Gunning, of New York, who used it nearly thirty years ago (February 12, 1861).

Dr. Bean, of Georgia, used it in the same manner at about the same time or a little later.

The fullest description of the appliance and its application is given by Dr. Gunning in the *New York Medical Journal* (between 1861 and 1867).

For a single, simple fracture of the lower jaw he employed a splint covering the lower teeth and gums, which he kept in place without fastening, the upper teeth resting upon the smooth upper surface, thus allowing free motion of the jaws, and causing but little inconvenience in speaking and eating. When found necessary, however, the splint was fastened by passing two or more screws through the rubber and into holes drilled in the teeth. In all fractures back of the teeth, Dr. Gunning advised a splint fitting over the crowns of the upper teeth as well as the lower, and so fastened by wings and straps that the lower jaw shall be held in a constant relation to the upper.

An apparatus constructed, it would seem, upon correct principles, but too complicated for general use, was devised by Dr. E. A. Clark and Dr. Homer Judd, of St. Louis. This consists of two separate plates fitted upon the upper and the lower teeth, with spiral springs between, and an elastic sling bandage passing under the jaw strong enough to counterbalance the action of the springs, and thus pressure and counterpressure are exerted upon the jaw to keep the fragments in position, while the whole is movable and under the control of the muscles.

I have recently been called upon to make and adjust the interdental splint in two cases, in each of which the lower jaw was broken through the body in the region of the right lateral incisor and through the left ramus.

The first was a merchant of Portland, who, on entering a railway station in a neighboring town, suddenly became faint or dizzy and fell forward, striking the face upon the corner of the door-step, inflicting severe bruises, breaking the lower jaw as described, and completely severing the superior maxillary bones from all connection with the bones of the skull. Just what the line of fracture was in the upper jaw it was impossible to determine; but it was probably through the body of each maxillary bone. This lesion was discovered while preparing to take the impression of the upper jaw, for, on grasping the upper teeth, the motion was quite free, the sensation being similar to that of taking hold of a very ill-fitting set of artificial teeth in the mouth.

The other case was that of a man employed in discharging coal from a vessel. A staging gave way and he fell to the deck.

One arm was broken, severe bruises inflicted, and the crown of the upper left central incisor broken off, in addition to the double fracture of the lower jaw. In each of these cases impressions were taken of the upper and the lower teeth without any attempt to reduce the fracture. Plaster models were made and the model of the lower jaw sawed through at the point of fracture, and adjusted in normal occlusion with that of the upper jaw. The two were then put in an articulator, and separated to a small extent to allow space between the front teeth for taking food. In one case a superior incisor had been previously lost, and in the other, one was broken at the time of the injury. This condition was somewhat convenient, but by no means necessary, in the treatment. After forming the splint in wax to cover the crowns of all the teeth, and to fill the space between the grinding surfaces of the teeth, the whole was removed from the articulator, flasked, packed, and vulcanized.

In finishing, the rubber was cut away in front, so that the upper front teeth were not covered, thus leaving a space. A narrow opening was also made at each side to allow the parotid saliva to flow into the mouth.

The splint was adjusted to the teeth of the upper jaw, and then the fragments of the lower jaw were brought into position, the teeth inserted in the sockets prepared for them, and the jaws kept closed for a time by means of the Garretson bandage. These were worn, in each case, about six weeks without removing, when the bone was found to be so well united that they were no longer needed.

